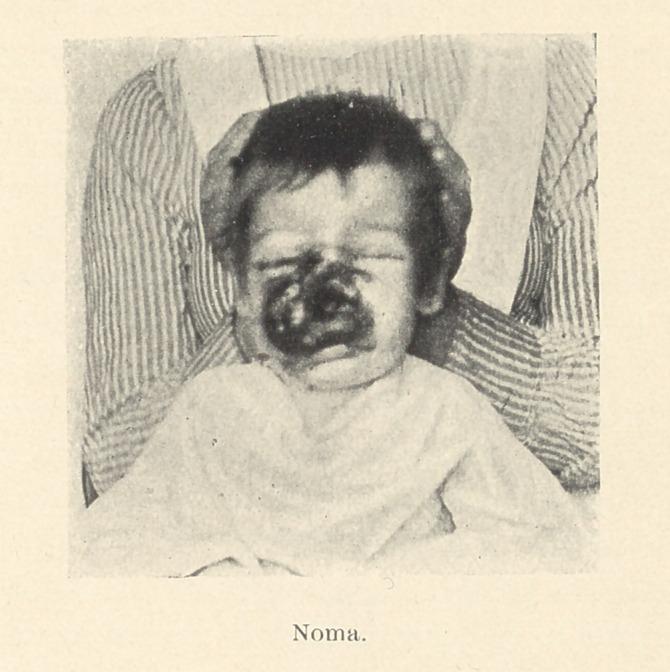# Noma, with Report of a Case

**Published:** 1903-06

**Authors:** Albert L. Midgley

**Affiliations:** Providence, R. I.


### NOMA, WITH REPORT OF A CASE.

BY ALBERT L. MIDGLEY, D.M.D., PROVIDENCE, R. I.[1]

NOMA, a disease of childhood, which is also known as cancrum oris, or gangrenous stomatitis, is a destructive, ulcerative condition of the mouth which makes its appearance upon the gum or mucous membrane of the cheek, and, advancing with great rapidity, destroys both soft and hard tissue and usually the life of the patient. It is essentially a secondary disease, and occurs most frequently during convalescence from measles, scarlatina, or other exhausting diseases of infancy. It sometimes arises, however, when these diseases have not been present, although it may be said that it never occurs in healthy children, but that it is almost invariably found in the weak, anæmic, poorly nourished type. It is more commonly found in the female, and appears most frequently in the lower jaw.

The cause of the disease is unknown, but many writers agree that the factors necessary for its appearance are a very low vitality and infection. Some say that it is of bacterial origin, and though many of the lower forms of life have been found, the reports as to their being the cause is a matter of opinion only. One micro-organism which is generally found is Lingard's thread-like bacillus. However, there seems to be no doubt that the disease is of an infectious nature, for several cases occurring in the same family have been reported. The excessive use of mercury favors its occurrence, and occasionally it has been found to follow an ulcerative stomatitis.

The onset of the disease is very insidious, and the most characteristic symptom is a very foul odor. Upon examination may be found an ashy gray ulcer on the mucous membrane of the gum or cheek, or at the angle of the mouth, which soon turns black. The cheek is swollen, tense, and shiny, and the skin, which is red, turns blue, and as the process advances a coal-black slough is left. A livid line marking the boundary of the diseased condition is also very characteristic. Other symptoms are an increased flow of saliva, drooling, coated tongue, diminished appetite, diarrhœa, and high temperature, though it may become subnormal before death.

---

[1] Assistant Dental Surgeon to St. Joseph's Hospital and Dental Externe to Rhode Island Hospital, Providence, R. I.

Noma.

The process very rarely becomes bilateral; relapses often occur, but the constitutional disturbance is very slight, and the child suffers little or no pain. A spontaneous recovery may take place even though there has been a great destruction of tissue, leaving a frightful deformity and impaired function of the jaws. The duration of the disease is from one to three weeks.

As to the pathology, Starr says that four distinct zones can be distinguished. Surrounding the destroyed tissue there is an infiltrated zone, then an area of increased connective tissue, and outside this is healthy tissue.

The prognosis of this disease is very grave. Seventy to ninety per cent. of the cases are fatal, and many believe that complications make the prognosis absolutely fatal. The child may die from septic pneumonia, diarrhœa, or septicæmia.

The curative treatment of noma lies in an early diagnosis and a prompt and radical operation. Isolation is absolutely necessary, and powerful disinfectants are to be made use of. Many believe in the free use of the Paquelin cautery and touching the parts with nitric acid or ninety-five per cent. carbolic acid. A stimulating tonic should be given, and the mouth should be kept in as hygienic condition as possible. Although the therapeutics cannot be said to have a specific character, chlorate of potash internally and as a mouth-wash is advised. After the operative stage has passed palliative measures only can be resorted to, and by attention to carious teeth and the various forms of stomatitis during convalescence from the exanthemata much can be done in the way of preventive treatment.

I report the following case:

R. K., aged two and a half years; male; seen at St. Vincent's Asylum. The patient presented the following history: His mother died of pulmonary phthisis. A healthy father and brother are still living. There was no history of children's diseases, and the patient had always enjoyed good health up to the present time.

*October 1, 1902.*—The patient was suffering from a dento-alveolar abscess of the right superior temporary lateral, and on this day I extracted the tooth, using chloride of ethyl as a local anæsthetic. Two days later there appeared at the gingival margin of the socket of the extracted tooth a black necrotic area, a quarter of an inch in diameter, which involved both soft tissue and bone. The skin of the upper lip on the right side was tense and waxy, and a distinct red discoloration of it marked the boundary of the inflammation. There was also a very foul odor, profuse ptyalism, and drooling. The tissues of the mouth were highly inflamed, and most of the teeth were in a very carious condition.

*October 4.*—Diagnosis, noma. I operated under chloroform anæsthesia and removed the roots of the superior temporary centrals and remaining lateral, since their crowns had been destroyed by caries. The necrosed tissue was entirely removed and the bone curetted and washed well with warm water and dioxygen. The wound was cauterized with carbolic acid ninety-five per cent. and packed loosely with gauze saturated with xeroform. Syrup of iodide of iron was given as a tonic and a mouth-wash of one per cent. silver nitrate was used every hour. The next day the patient appeared very comfortable, with normal temperature and pulse, and no pain apparently.

The following day, to my surprise, the roof of the mouth, floor of the nose, and half of the right cheek were in a gangrenous condition and beyond the stage of a second operation. From now until death, October 19, palliative measures only were used. The temperature during these days was 102° to 103° F. and the pulse ranged from 120 to 140 per minute. Diarrhœa began the second day after the operation and continued throughout the disease. A specimen was given to the pathologist of the hospital for examination, and he found a thread-like bacillus.

The case is of interest, since the disease immediately followed a dento-alveolar abscess and attacked a strong healthy child. Another important point also to be considered is that two weeks previously a child died from the same disease in this institution.

BIBLIOGRAPHY.

System of Oral Surgery (Garretson), page 498.
Treatise on Surgery (Moullin), page 720.
System of Surgery (Dennis), vol. iii. page 127.
Medical Record, vol. xii.
Surgical Diseases of Children (Owen), page 193.
International Encyclopædia of Surgery, vol. v. page 495; vol. ii. page 308.
American Text-Book of the Diseases of Children (Starr).
International Text-Book of Surgery (Warren and Gould), page 230.
American Text-Book of Surgery, pages 53, 646.
Treves' Surgery.
Holt's Surgery.

## Figures and Tables

**Figure f1:**